# Peroxisome proliferator-activated receptor γ agonist effect on rheumatoid arthritis: a randomized controlled trial

**DOI:** 10.1186/ar4290

**Published:** 2013-09-10

**Authors:** Michelle J Ormseth, Annette M Oeser, Andrew Cunningham, Aihua Bian, Ayumi Shintani, Joseph Solus, S Bobo Tanner, C Michael Stein

**Affiliations:** 1 Division of Rheumatology, Department of Medicine, Vanderbilt University, Nashville, TN, USA; 2 Division of Clinical Pharmacology, Department of Medicine, Vanderbilt University, Nashville, TN, USA; 3 Department of Biostatistics, Vanderbilt University, Nashville, TN, USA

## Abstract

**Introduction:**

Rheumatoid arthritis (RA), a chronic inflammatory disease, is associated with insulin resistance. Experimental evidence indicates that the relationship between insulin resistance and inflammation is bidirectional: Inflammation promotes insulin resistance, and insulin resistance promotes inflammation. Therefore, we examined the hypothesis that pioglitazone, a thiazolidinedione peroxisome proliferator-activated receptor γ agonist, would decrease inflammation and disease activity and improve insulin resistance in patients with RA.

**Methods:**

In a single-center, randomized, double-blind, placebo-controlled crossover study patients with RA (*N* = 34) receiving stable therapy were randomized to also receive either pioglitazone 45 mg daily (*n* = 17) or matching placebo (*n* = 17) for eight weeks. This was followed by a four-week washout period and alternative treatment for eight weeks. Outcomes included change in Disease Activity Score in 28 joints (DAS28) score, individual components of the DAS28 score and homeostatic model assessment for insulin resistance (HOMA). Intention-to-treat analysis and linear mixed-effects models were used.

**Results:**

Patients had a mean (±SD) age of 51 (±14.2) years, 82.4% were female and baseline DAS28 high-sensitivity C-reactive protein (DAS28-CRP) was 4.58 (±1.1) units. Addition of pioglitazone was associated with a 9.3% reduction (95% confidence interval (CI) = 0.17% to 17.6%) in DAS28-CRP (*P* = 0.046), but no significant change in DAS28 erythrocyte sedimentation rate (DAS28-ESR) (*P* = 0.92). There was a 10.7mm (95% CI = 0.4 to 20.9 mm) improvement in patient-reported global health (*P* = 0.042), a 48.6% decrease (95% CI = 27.6% to 63.5%) in CRP (*P* < 0.001) and a 26.4% decrease (95% CI = 3.7% to 43.8%) in insulin resistance as measured by HOMA (*P* = 0.025), but no significant reduction in swollen or tender joint count or in ESR (all *P* > 0.05). Lower-extremity edema was more common during pioglitazone treatment (16%) than placebo (0%).

**Conclusion:**

Addition of pioglitazone to RA therapy improves insulin resistance and modestly reduces RA disease activity measured by DAS28-CRP and two of its components, including patient-reported global health and CRP, but not DAS28-ESR or ESR.

**Trial registration:**

NCT00763139

## Introduction

Patients with rheumatoid arthritis (RA), a chronic systemic inflammatory disease, have an increased incidence of premature atherosclerosis, even after adjusting for traditional cardiovascular risk factors [1]. We have reported previously that patients with RA have more than a twofold increased incidence of insulin resistance and that this was associated with inflammation, increased disease activity and coronary atherosclerosis [2,3]. Moreover, insulin resistance has been associated with increased inflammation in populations without RA [4-8]. Additional evidence that inflammation can promote insulin resistance is provided by studies in animals and humans [9-11]. For example, in diabetic rats, administration of tumor necrosis factor α (TNF-α) caused hyperglycemia [12], and, in obese rats, neutralization of TNF-α improved insulin resistance [13]. In humans, treatment of active RA, even with corticosteroids, a class of drug which would be expected to increase insulin resistance, can decrease insulin resistance, presumably by decreasing inflammation [14].

Recent evidence indicates that, just as inflammation may result in insulin resistance, hyperinsulinemia may increase inflammation. For example, *in vitro* studies have shown that glucose and insulin-like growth factors augment the secretion of inflammatory cytokines in mononuclear cells [15,16]. Also, in humans, euglycemic hyperinsulinemia amplifies and prolongs the interleukin 6 (IL-6) response to endotoxin [17]. Thus, there appears to be a bidirectional relationship between inflammation and hyperinsulinemia.

Concordant with the finding that insulin resistance may promote inflammation, therapies which improve insulin resistance may decrease inflammation. Thiazolidinediones, selective ligands of the nuclear transcription factor peroxisome proliferator-activated receptor γ (PPAR-γ), are insulin sensitizers, even in individuals without diabetes [18]. Studies in animals, including a model of arthritis, as well as in patients with psoriatic arthritis and lupus, have shown that, in addition to improving insulin sensitivity, thiazolidinediones decrease inflammation [19-28].

We therefore performed this study to test the hypothesis that pioglitazone would decrease disease activity, inflammation and insulin resistance in patients with RA.

## Methods

### Patients

Patients older than 18 years of age who met the 1987 American Rheumatism Association criteria for RA [29] with moderate disease activity (at least three tender and three swollen joints) and no change in immunomodulating or anti-inflammatory therapy in the past one month were eligible for enrollment. Patients were excluded if they had active cancer other than skin cancer, HIV, heart failure, severe edema, diabetes mellitus requiring drug therapy, aspartate aminotransferase or alanine aminotransferase greater than twice the upper limit of normal, major surgery within the previous three months, untreated osteoporosis, allergy to pioglitazone, previous organ or bone marrow transplant or severe comorbid conditions likely to compromise survival or study participation. Patients were also excluded if they were receiving dialysis, taking gemfibrozil or rifampin or unwilling or unable to cooperate. Additionally, patients were excluded they were pregnant, or if they had childbearing potential and would not agree to affective birth control. After reviewing reports about the possible association between pioglitazone and bladder cancer [30,31], we excluded patients with a history of bladder cancer or precancerous bladder lesions. The study was approved by the Vanderbilt University Institutional Review Board, and all participants gave their written informed consent.

### Study design

**Figure 1:**
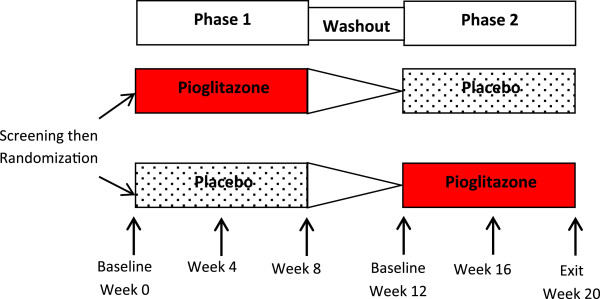
**Randomized, placebo-controlled, crossover trial design.** Patients were screened for eligibility. Those meeting entry criteria were randomized to one of two treatment sequences: pioglitazone then placebo or placebo then pioglitazone. Patients were assessed every four weeks. Treatment phases were separated by a four-week washout period.

This trial was a single-center, randomized, double-blind, placebo-controlled crossover study with washout. We chose to use a crossover study design because this model increases the power to detect a difference with small sample sizes by reducing between-patient variability and the effect of confounders when each patient serves as his or her own control. Patients were randomly assigned to receive pioglitazone 45 mg or matching placebo daily for eight weeks in combination with unchanged doses of their baseline disease-modifying antirheumatic drug (DMARD) therapy. This treatment was followed by a four-week washout period, then a second eight-week treatment period with the alternative therapy (Figure 1). Study visits occurred at screening, at baseline (week 0) and every four weeks through week 20 or withdrawal from the study. Patients were randomly assigned to a treatment sequence (placebo or pioglitazone first) according to a randomization table generated using a permuted block randomization scheme prepared by the study statistician. Pioglitazone or identical placebo capsules were dispensed by the Vanderbilt University investigational pharmacy. If a change in RA disease status warranted any change in DMARD or corticosteroid therapy, the patient was withdrawn from the study.

At the baseline (week 0) visit, the following clinical details were obtained from the patient and the medical records: height, weight, waist and hip measurements; blood pressure; current and past smoking history; personal and family history of cardiovascular disease; medication use, including current and past DMARD use; current and cumulative prednisone therapies; and cardiovascular therapies.

At baseline and at each subsequent visit, participants were asked to temporarily omit medications, including nonsteroidal anti-inflammatory drugs (NSAIDs) and corticosteroids, on the morning of the study day. Patients were studied after at least 6 hours of fasting and after 30 minutes of supine rest at approximately the same time of day. A physical examination was performed, and RA disease activity indices, including 28 tender and swollen joint counts, were measured and venous blood was drawn for laboratory assessments. Urine and/or serum pregnancy tests were performed at each study visit in females with reproductive potential.

Laboratory examinations comprising a complete blood count; creatinine, glucose and liver function tests (LFTs); high-sensitivity C-reactive protein (CRP) and erythrocyte sedimentation rate (ESR) were performed by the hospital clinical laboratory staff. Fasting plasma insulin levels were measured at the Vanderbilt Hormone Assay Core Laboratory by radioimmunoassay. Serum cytokines, including tumor necrosis factor α (TNF-α) and IL-6, were measured by multiplex enzyme-linked immunosorbent assay (EMD Millipore, Billerica, MA, USA) in duplicate in batches with samples from each time point in a given patient run together on the same plate with appropriate controls and blocking agents. All measurements were taken at the beginning and end of each treatment arm.

### Outcome measures

The primary outcome was change in Disease Activity Score in 28 joints (DAS28) score (calculated as DAS28-CRP = 0.56 * sqrt(tender_28_) + 0.28 * sqrt(swollen_28_) + 0.70 * ln(CRP) + 0.014 * global health (GH) and DAS28-ESR = 0.56 * sqrt(tender_28_) + 0.28 * sqrt(swollen_28_) + 0.70 * ln(ESR) + 0.014 * GH). Other prespecified outcomes included individual components of the DAS28 score, including tender and swollen joint count, patient-reported GH based on 1- to 100-mm Visual Analogue Scale (VAS) rating and acute-phase reactants (CRP, ESR); level of inflammatory cytokines IL-6 and TNF-α; change in functional capacity determined using the modified Health Assessment Questionnaire (MHAQ) [32]; and change in insulin resistance determined by the homeostatic model assessment for insulin resistance (HOMA), calculated as (fasting glucose [in mmol/L] × fasting insulin [in μU/ml])/22.5 [33].

### Statistical analysis

Sample size was estimated using a paired *t*-test and assuming a baseline DAS28 score of 5.0 ± 1.0 units [34,35]. A sample size of 32 patients would provide 90% power to detect a 0.6-unit greater decrease in DAS28 score in patients taking pioglitazone compared to placebo after excluding dropouts (defined as having no follow-up study visits). We initially aimed to enroll 36 patients with the assumptions that 4 patients would drop out (defined as not returning for any visits) and 32 patients would provide data for analysis. We stopped enrollment after patient 34, however, because there had been no dropouts.

Descriptive statistics are presented as frequencies and percentages (%) for categorical variables and means with standard deviations (mean ± SD) for continuous variables. Linear mixed-effects models were used to quantify the effect of pioglitazone on outcomes. Data gathered from all study visits from all patients, including those who exited the study early and those who were withdrawn, were used in the analysis. The linear mixed-effects models were fitted for all outcomes separately. These modeled the fixed effects of the baseline value of the outcome, treatment regimen, week and product of week and treatment regimen. Random intercepts were used to model the variability between patients. For all the primary and secondary outcomes, the carryover effect was assessed by comparing the means of the baseline outcome measurements between patients who received pioglitazone in the first phase followed by placebo and those who received placebo in the first phase followed by pioglitazone using linear mixed-effects models with random intercepts for between-patient variations. Because we used a two-arm crossover design in this study, data were pooled between the two treatment phases after finding no carryover effect, and we report between-group differences at the end of study time points (week 8 and week 20) using general contrast of regression coefficients in the mixed-effects models.

To assess whether pioglitazone’s effect on HOMA mediated the effect on disease indices such as DAS28-CRP, we used mixed-effect models to examine attenuation in the effect of the treatment by adjustment for the change in HOMA [36]. The values for DAS28-CRP, DAS28-ESR, CRP, ESR, MHAQ, IL-6, TNF-α and HOMA were all natural logarithm–transformed to improve normality in the residuals. Thus, changes in these outcomes were back-transformed and presented as percentage changes. Statistical analyses were performed using the R Project for Statistical Computing version 2.15.1 software (http://www.r-project.org/). A two-sided significance level of 5% was required to consider the data as statistically significant.

## Results

### Patients

**Figure 2:**
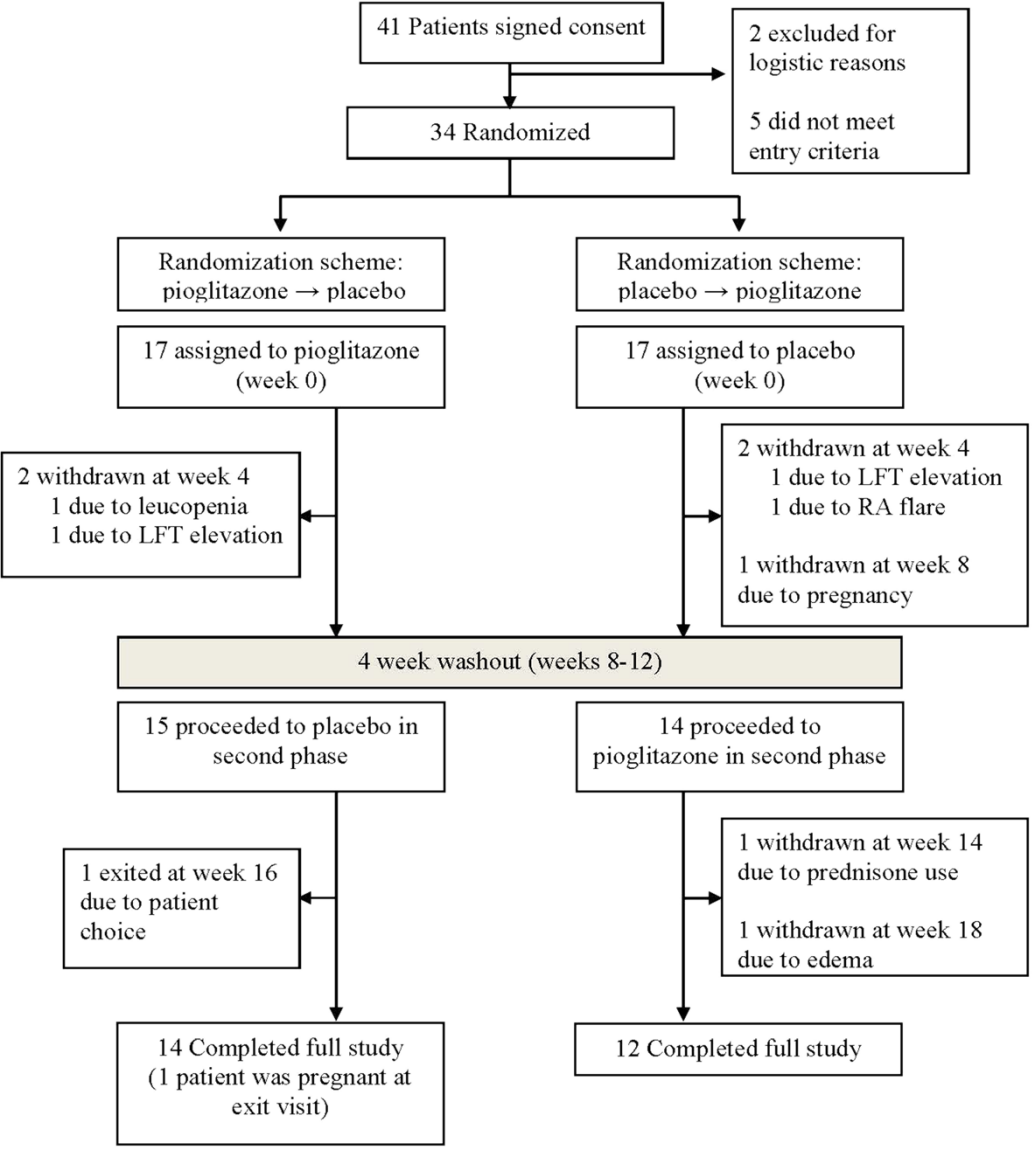
**Randomization and follow-up of study participants.** A total of 34 patients with rheumatoid arthritis (RA) were enrolled in the study. There was at least one follow-up visit for all patients. A total of 31 patients were exposed to pioglitazone, and 32 were exposed to placebo. LFT, liver function test.

The study was performed from May 2009 to April 2012. Thirty-four patients with RA were studied (Figure 2). Patients had a mean age (±SD) of 51.0 years (±14.2), 82.4% were female and the baseline mean DAS28-CRP was 4.58 units (±1.1). Of the 28 patients with available rheumatoid factor (RF) or anti-citrullinated peptide antibody (ACPA) test results, 24 (85.7%) were RF- or ACPA-positive. Of the 21 patients with X-rays, 15 (71.4%) had erosive disease. The baseline characteristics of the patients are shown in Table 1.

**Table 1:** **Baseline demographics and clinical characteristics of patients**^**a**^

**Demographics and characteristics**	**Data**
Demographics and anthropomorphic measures	
Age (years)	51.0 (±14.2)
Females (%)	82.4% (28)
Race, Caucasians (%)	85.3% (29)
Weight (kg)	77.3 (±18.1)
BMI (kg/m^2^)	28.28 (±6.04)
Disease-related and laboratory indices	
DAS28-CRP (units)	4.58 (±1.10)
Tender joints (*n*)	9.68 (±6.96)
Swollen joints (*n*)	8.38 (±4.66)
VAS (mm)	43.0 (±28.5)
CRP (mg/dl)	12.69 (±24.57)
ESR (mm/h)	22.8 (±21.3)
HOMA (units)	3.11 (±2.68)
Current medication use	
Methotrexate (%)	70.6% (24)
Leflunomide (%)	14.7% (5)
Sulfasalazine (%)	2.9% (1)
Hydroxycholorquine (%)	17.6% (6)
Biologic (%)	52.9% (18)
Corticosteroid (%)	52.9% (18)

^a^Data are presented as mean (±SD) for continuous data and percentage (number) for categorical variables. BMI, body mass index; CRP, high-sensitivity C-reactive protein; DAS28-CRP, Disease Activity Score in 28 joints high-sensitivity C-reactive protein; ESR, erythrocyte sedimentation rate; HOMA, homeostatic model assessment of insulin resistance; VAS, Visual Analogue Scale for Global Health.

### Effect of pioglitazone on rheumatoid arthritis disease indices and inflammatory markers

**Figure 3:**
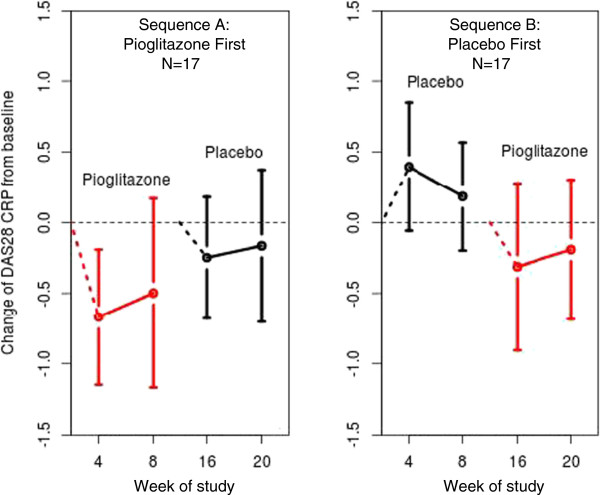
**Rheumatoid arthritis disease activity measured by Disease Activity Score in 28 joints score is decreased by pioglitazone.** The graph on the left shows the change in Disease Activity Score in 28 joints high-sensitivity C-reactive protein (DAS28 CRP) scores among patients randomized to receive pioglitazone first, then placebo. The graph on the right shows change in DAS28-CRP scores among patients randomized to receive placebo first, then pioglitazone. Red designates patients taking pioglitazone, and black represents those taking placebo. Open circles represent mean change, and bars represent 95% confidence intervals.

The addition of pioglitazone was associated with a 9.3% decrease (95% confidence interval (95% CI) = 0.17% to 17.6%) in DAS28-CRP (*P* = 0.046) compared to placebo; however, the effect of pioglitazone on DAS28-ESR was not statistically significant (*P* = 0.92) (Table 2 and Figure 3). Individual components of the DAS28 score were evaluated. Pioglitazone was associated with a 10.7-mm improvement (95% CI = 0.4- to 20.9-mm) in patient-reported GH on the VAS (*P* = 0.042) and a 48.6% decrease in CRP (95% CI = 27.6% to 63.5%) (*P* < 0.001), but no significant change in swollen joint count (*P* = 0.77), tender joint count (*P* = 0.54) or ESR (*P* = 0.32) compared to placebo (Table 2). There was no significant change in MHAQ score (*P* = 0.51) with pioglitazone use (Table 2). Use of pioglitazone was associated with a 67.0% reduction in IL-6 (95% CI = 28.2% to 84.6%) (*P* = 0.01), but no significant change in TNF-α (*P* = 0.71) (Table 2).

**Table 2:** **Effect of pioglitazone on rheumatoid arthritis disease activity, inflammation and insulin resistance**^**a**^

**Measurement**	**Pioglitazone phase**		**Placebo phase**		**Pioglitazone treatment effect**	
	**Baseline**	**Week 8/week 20**	**Baseline**	**Week 8/week 20**	**Effect**	***P*-value**
	**(SD)**	**(SD)**	**(SD)**	**(SD)**	**(95% CI)**	
DAS28-CRP	4.40	4.03	4.57	4.48	−9.3%	0.046
	(1.00)	(1.15)	(1.28)	(1.20)	(−17.6 to −0.17%)	
DAS28-ESR	4.56	4.37	4.85	4.57	0.6%	0.92
	(1.39)	(1.28)	(1.56)	(1.63)	(−10.3 to 12.7%)	
Swollen joints (*n*)	6.6	6.46	8.2	7	0.3	0.77
	(3.7)	(4.79)	(5.6)	(5.11)	(−1.4 to 1.9)	
Tender joints (*n*)	9.6	8.54	11.5	10.35	−0.7	0.54
	(6.89)	(7.28)	(8.1)	(8.23)	(−2.9 to 1.5)	
VAS (mm)	44.9	42.04	48.0	49.46	−10.7	0.042
	(24.7)	(28.39)	(31.2)	(23.62)	(−20.9 to −0.4)	
CRP (mg/dl)	8.1	5.02	7.7	8.25	−48.6%	<0.001
	(11.41)	(7.64)	(13.6)	(10.32)	(−63.5 to −27.6%)	
ESR (mm/h)	18.5	17	19.5	18.88	21.3%	0.32
	(18.2)	(17.06)	(20)	(20.80)	(−16.9 to 77%)	
IL-6 (pg/ml)	5.41	2.38	8.67	6.47	−67.0%	0.01
	(7.88)	(4.05)	(19.31)	(14.40)	(−84.6 to −28.2%)	
TNF-α (pg/ml)	9.90	9.50	13.40	9.71	6.0%	0.71
	(10.64)	(10.56)	(19.41)	(7.91)	(−22.7 to 46.2%)	
MHAQ (units)	0.55	0.54	0.56	0.59	−4.3%	0.51
	(0.37)	(0.39)	(0.35)	(0.36)	(−16.2 to 9.3%)	
HOMA (units)	2.83	2.44	2.38	3.11	−26.4%	0.025
	(2.50)	(2.08)	(1.75)	(3.47)	(−43.8 to −3.7%)	

^a^Data for each intervention phase (pioglitazone and placebo) at baseline and at week 8/week 20 are presented as mean (±SD). Pioglitazone treatment effect was calculated using linear mixed-effects models and is presented as change or percentage change (95% confidence interval). CRP, high-sensitivity C-reactive protein; DAS28-CRP, Disease Activity Score in 28 joints high-sensitivity C-reactive protein; ESR, erythrocyte sedimentation rate; HOMA, homeostatic model assessment of insulin resistance; IL-6, interleukin 6; MHAQ, modified Health Assessment Questionnaire; TNF-α, tumor necrosis factor α; VAS, Visual Analogue Scale for Global Health.

### Effect of pioglitazone on insulin resistance and relationship to disease indices

Use of pioglitazone was associated with a 26.4% decrease (95% CI = 3.7% to 43.8%) in insulin resistance as measured by HOMA (*P* = 0.025) (Table 2). To determine whether improvement in insulin resistance caused the improvements in disease activity indices, we compared the results of our above-described models with the results after additional adjustment for HOMA. There was no attenuation of effect after adjustment for HOMA: DAS28-CRP, −9.8% (95% CI = −18.1% to –0.6%; *P* = 0.037); CRP, −48.9% (95% CI = −66.2 to −22.8%; *P* = 0.001); VAS, 10.5 mm (95% CI = −19.6 to −1.35; *P* = 0.002); and IL-6, −71% (95% CI = −87.8 to −31.8%; *P* = 0.005). Thus, we determined that there was no statistical evidence that pioglitazone’s effect on HOMA mediated changes in outcome measures of inflammation.

### Drug side effects

Treatment with pioglitazone was generally well-tolerated. Adverse effects related to the drug included lower-extremity edema in five patients (16%) compared to none among those taking placebo. Four patients were withdrawn during the pioglitazone arms of the study (one each due to leukopenia, LFT elevation, new prednisone use and lower-extremity edema). Similarly, four patients were withdrawn during the placebo arms of the study (one each due to LFT elevation, RA flare, unintended pregnancy and patient choice) (Figure 2). Use of pioglitazone was associated with a 0.76 kg increase (95% CI = 0.10 to 1.42 kg) in body weight (*P* = 0.023).

### Washout and assessment for carryover effect

There was no carryover effect detected for DAS28-CRP (*P* = 0.92) or other outcomes evaluated (all *P* > 0.05).

## Discussion

The major findings of this study are that the addition of pioglitazone to stable DMARD treatment for RA improves insulin resistance, as measured by HOMA, and modestly reduces RA disease activity, as measured by DAS28-CRP, patient-reported GH and CRP levels. The reductions in DAS28-CRP, CRP and IL-6 levels were independent of improvement in insulin resistance.

Several lines of evidence support the idea that PPAR-γ agonists have anti-inflammatory effects. PPAR-γ expression in peripheral monocytes and monocyte-derived macrophages in RA patients was inversely correlated with disease activity, and some medications commonly used in the treatment of RA (methotrexate and corticosteroids) increase PPAR-γ expression [37]. In animal models of RA, thiazolidinedione treatment reduced synovitis, inflammatory bone loss and bone erosion [23-25]. Moreover, a recent single-blind trial suggested improvement in disease activity in diabetic patients with active RA, but an adequate comparator control was not used; thus, definitive conclusions regarding efficacy could not be made [25]. Additionally, in an open-label trial of patients with psoriatic arthritis, pioglitazone reduced both tender and swollen joint counts [26], and pioglitazone decreased CRP and serum amyloid A and improved insulin sensitivity in patients with uncomplicated systemic lupus erythematosus [27].

Our findings raise the possibility that pioglitazone may have different effects on CRP compared to ESR. CRP is produced in the liver in response to IL-6 [38], whereas ESR is a laboratory phenomenon affected by fibrinogen, immunoglobulin levels and red blood cell count and shape, among other factors [39,40]. Several studies have found that pioglitazone decreased CRP concentrations [19-21], but ESR has not been measured routinely in most clinical trials of pioglitazone or other thiazolidinediones. However, in one study that included nondiabetic patients with known cardiovascular disease, pioglitazone resulted in a nonsignificant trend toward increased ESR [41]. The decrease in IL-6 concentrations we observed after pioglitazone therapy is mechanistically concordant with the reduction in CRP that occurred, but factors driving ESR may not have been altered as much [42,43]. In contrast to IL-6, TNF-α concentrations were not significantly affected by pioglitazone, raising the possibility that pioglitazone may have greater effects on IL-6 than on TNF-α. These modest effects of pioglitazone suggest it is unlikely to be a helpful add-on therapy for reduction of RA disease activity, particularly when one considers the increased risk for fracture, bladder cancer and heart failure in some individuals [44-46].

As part of our analysis, we set out to define whether changes in insulin sensitivity were related to changes in inflammation because this would provide a mechanistic insight. We found that improvements of DAS28-CRP, CRP and IL-6 after pioglitazone treatment were independent of improvements in HOMA, suggesting that mechanisms other than improved insulin sensitivity are likely to be involved. These findings are compatible with those of a study of healthy participants showing that rosiglitazone reduced CRP within one day, before changes in insulin sensitivity had occurred [47]. Thus, thiazolidinediones may affect inflammation rapidly, before the effects on insulin resistance have occurred, through mechanisms independent of improved insulin sensitivity [47-51].

Our study has some limitations. This study was relatively small, but it was efficiently designed to study the outcomes of interest. The crossover design has the advantage of comparing changes in the same patient and the disadvantage that subtle, undetected carryover effects may have occurred. Several mean baseline disease activity indices (DAS28-CRP, DAS28-ESR, swollen and tender joint counts, VAS, IL-6 and TNF-α) were slightly lower at the start of the pioglitazone phase compared to the baseline of the placebo phase. Though not a significant difference, lower disease activity in patients entering the treatment phase could have biased our results toward the null hypothesis. Also, inflammatory cytokine levels were relatively low even at baseline because patients were receiving fairly effective background therapy, which also could bias our results toward the null hypothesis. Duration of drug exposure was only eight weeks, which is shorter than most trials of DMARDs and biologics; therefore, we cannot exclude the possibility that extended exposure to pioglitazone may have had different effects on joint counts and overall disease activity.

## Conclusion

Addition of pioglitazone to RA therapy improves insulin resistance and modestly reduces RA disease activity measured by DAS28-CRP and two of its components, patient-reported GH and CRP, but not DAS28-ESR or ESR.

## Abbreviations

ACPA: anti-citrullinated peptide antibody; CRP: high-sensitivity C-reactive protein; DAS: Disease Activity Score; DMARD: disease-modifying antirheumatic drug; ESR: erythrocyte sedimentation rate; GH: global health; HOMA: homeostatic model assessment of insulin resistance; IL-6: interleukin 6; LFT: liver function test; MHAQ: Modified Health Assessment Questionnaire; NSAID: nonsteroidal anti-inflammatory drug; PPAR-γ: peroxisome proliferator-activated receptor γ; RA: rheumatoid arthritis; RF: rheumatoid factor; SD: standard deviation; TNF-α: tumor necrosis factor α.

## Competing interests

The authors declare that they have no competing interests.

## Authors’ contributions

MJO performed patient recruitment, study execution, data analysis and interpretation, and drafted the manuscript. AMO and AC performed patient recruitment and study execution. AB performed data analysis. AS participated in study design and performed data analysis. JS performed enzyme-linked immunosorbent assays. SBT performed patient recruitment. CMS conceived the study, established the study design, oversaw study execution, performed data interpretation and oversaw manuscript drafting. All authors read and approved the final manuscript.
